# Energy Absorption Capacity of SBR Latex-Modified Ordinary Portland Cement by Charpy Impact Test

**DOI:** 10.3390/ma14102544

**Published:** 2021-05-13

**Authors:** Tri N. M. Nguyen, Jung J. Kim

**Affiliations:** Department of Civil Engineering, Kyungnam University, Changwon-si 51767, Korea; nnmtri@utc2.edu.vn

**Keywords:** energy absorption capacity, SBR latex, Charpy impact test, compressive strength, Weibull distribution

## Abstract

The present study deals with tests on the energy absorption capacity and compressive strength of styrene–butadiene rubber (SBR) latex-modified cementitious materials. Different polymer–cement ratios (P/C) of 0, 5, 10, 15, and 20% were carried out with the Charpy impact test at 7, 14, and 28 days of curing. The observations showed an increase in the energy absorption capacity of the SBR latex-modified cement paste in correspondence with the increase in curing times, as well as the increase in the P/C ratios. The P/C ratio of 10% was the optimal ratio for observing the highest energy absorption capacity of the SBR latex-modified cement paste, with a 43% increase observed. In addition, a linear relationship between compressive strength and the energy absorption capacity at 28 days was proposed. Based on that, the energy absorption capacity of SBR latex-modified cement paste can be analyzed or predicted by the compressive strength results, regardless of the P/C ratios. Finally, the two-parameter Weibull distribution was proved to fit by the observation data from the Charpy impact test.

## 1. Introduction

Cement has long been viewed as a widely used material in the construction industry that plays a role as an essential binder in the composition of concrete [1,2]. The failure of concrete in general, and of cement in particular, is an undesirable phenomenon for several different reasons, the most obvious of this being the potential effect on impact loading. Experiments such as the explosive test [3], the projectile impact test [4], the drop-weight impact test [5], and the split Hopkinson pressure bar test [6] have been created to ascertain the dynamic mechanical properties of materials. Among these tests, the Charpy pendulum impact test is most commonly used to evaluate the toughness of the energy absorption capacity of materials [7]. This experiment determines the energy absorbed during the fracturing of materials. It utilizes small-sized specimens and a simple pendulum impact tester, and therefore can be conducted quickly and easily compared to other standard fracture toughness tests, and is much cheaper to use. It is not only utilized and standardized for testing metallic materials (ASTM E23, ISO 148) [8,9] or plastic materials (ASTM D6110, ISO 179) [10,11], but also is applied to composites, ceramics, and polymers [12,13,14]. In particular, the literature review shows the energy absorption capacity of cement-based materials as measured by the Charpy pendulum impact test [15]. Hakamy et al. [16] found good interfacial bonding between the fibers and nanomatrix, and observed an increase of 23% in the impact strength of a hemp fabric-reinforced nanoclay–cement composite compared to that of a hemp fabric-reinforced cement composite. The work [17] showed through the Charpy impact test the decrease in resistance of a glass fiber-reinforced cement containing an acrylic polymer emulsion due to the emulsion reducing the elastic modulus of the cement-based material. In addition, the energy absorption capacity of ultra-high-performance hybrid fiber reinforced concrete has been examined by Yu et al. [18]. They found the dominating role that long steel fiber has in improving the Charpy impact resistance of that material.

From another perspective, improving the performance of cement-based materials by a polymer admixture has been conducted since the 1920s [19,20]. The observations showed the positive influence polymer has on improving strength, deformability, workability, durability, and so on. From microstructural investigations, the improving mechanism of the polymer was due to the incorporation of the cement hydration process and polymer film formation in the matrix phase. The work of Ohama [21] found the chemical reactions between polymer particles and calcium-based and silicate-based components of cement. These reactions were expected to improve the bond between the binder and aggregates. In addition, the formation of polymer films showed a positive effect on bridging the microcracks in the matrix under the stressed conditions, leading to a restriction in the propagation of cracks. Overall, these reactions improve the performance of cement-based materials. The literature review showed numerous types of polymers such as styrene–acrylate polymer, copolymer of vinyl propionate and vinylidene chloride, acrylate with a coupling agent polymer, ethylene–vinyl acetate copolymer, ethylene–acetate ethylene interpolymer, and so on, have been utilized to improve cement-based materials [22,23,24]. Recently, styrene–butadiene rubber (SBR) latex has been considered as a beneficial polymer for improving the performance of cement-based materials. From the microstructural perspective, the work [25] found the filling effect of SBR latex led to improving the degree of density of the interfacial transition zone (ITZ). From the cement hydration process perspective, the works [26,27,28] found the retardation effect of SBR latex on the cement hydration process at an early age, but this inhibiting effect becomes weaker with a long curing period. In addition, the degree of cement hydration decreases with the increase in SBR solid/water ratio up to 20%. From the physical behavior perspective, the fluidity of the cement paste containing SBR latex increased with the increase in latex content [29,30]. From the mechanical properties perspective, the observations in the literature showed the decrease in compressive and flexural strengths corresponded with the increase in SBR latex content [31,32]. It is worth mentioning that the polymers themselves exhibited a high impact resistance, therefore, latex-modified cement-based materials were recognized with a good energy absorption capacity compared to the conventional ones [21]. As reviewed above, there are many benefits of SBR latex in regard to improving cement-based materials. However, studies on the energy absorption capacity of SBR latex-modified cement paste have not been carried out. In this study, the energy absorption capacity of SBR latex-modified cement paste was examined by use of the Charpy impact test. Different polymer–cement ratios (P/C) of 0, 5, 10, 15, and 20%, as well as different curing times of 7, 14, and 28 days, were considered in this work. In addition, the compressive strength of SBR latex-modified cement paste at 28 days was observed to find the relationship between the compressive strength and the energy absorption capacity of these modified cementitious materials. Finally, the statistical approach was utilized to clarify the observations from the experimental test and the effect of SBR latex on the energy absorption capacity of cement paste.

## 2. Experimental Section

### 2.1. Materials and Specimens

Materials in this study included ordinary Portland cement (Ssangyong Co., Seoul, Korea), type I, compliance with ASTM C150/150M [33], and an emulsion form of sstyrene–butadiene rubber (SBR) latex (JAPT-1520, Jung Ang Polytech Co., Gyeongsangnam, Korea). The physical properties of SBR latex are summarized in Table 1.

The cement paste specimens were prepared with the constant water–cement ratio of 0.4, and the polymer–cement ratio (P/C) of 0, 5, 10, 15, and 20%, respectively. It is worth mentioning that the total solid content (TSC) of latex is 47%, therefore, when determining the amount of SBR latex polymer for the experiment, this factor needed to be considered. The mixture designs are presented in Table 2, in which names of the specimens are marked from SBR00 to SBR20, in that order, for the P/C ratios from 0% to 20%.

**Figure 1:**
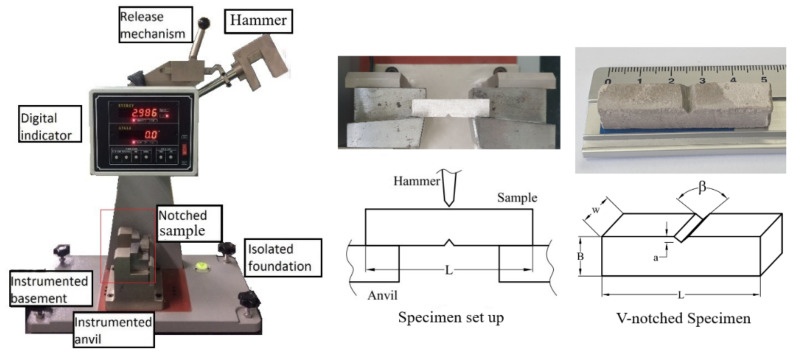
The Charpy impact test.

All of the specimens were cured in the water under laboratory conditions (20 ± 20 °C, 50 ± 5% R.H). The Charpy specimen was proposed in the form of a 10 × 10 × 50 mm v-notched bar [15]. The specimen dimensions are clarified in Figure 1, where β = 45°–90°, a = 2 mm, w = B = 10 mm, L = 50 mm. The tests were conducted after 7, 14, and 28 days of curing.

**Figure 2:**
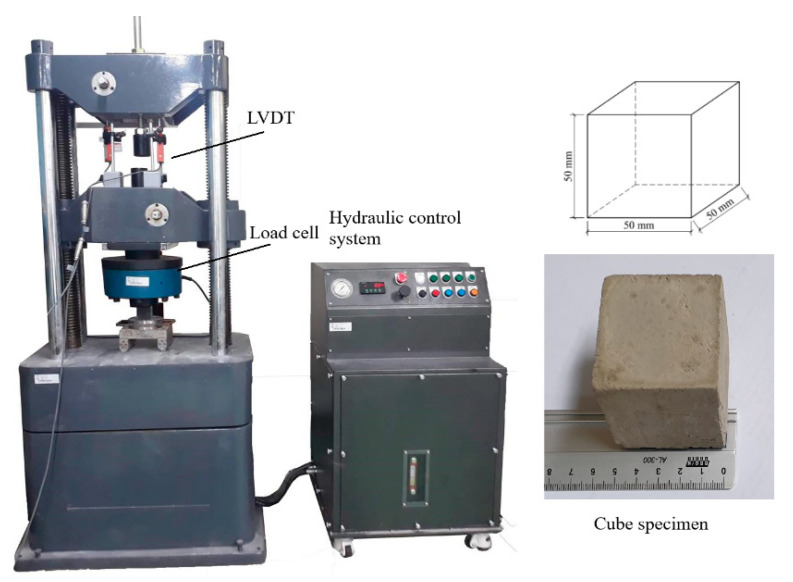
The compressive strength test.

The specimen for the compressive strength test was a 50 × 50 × 50 mm cube (see Figure 2), complying with the specification of ASTM C 109/109M [34], and the test was conducted at the age of 28 days.

### 2.2. Testing Methods

The breaking energy of hardened cement paste specimens was observed by the Charpy pendulum impact tester MT-333 (Dong Ah Testing Machine Co., Seoul, Korea). At the initial state, a 0.7 kg pendulum hammer was attached to the machine body using a pinned rotating arm at a height of 0.435 m, and with a stored energy of 2.986 J. The specimen was supported at both ends by two anvils parted like a simple beam. The fracture occurs after the falling hammer hits, with impacts on the face opposite the notch. It is worth noting that if the pendulum is stopped by the specimen, i.e., the fractured impact specimen does not separate into two parts, the pendulum mass should be increased and the test reconducted [11]. The energy transferred to the material can be inferred by comparing the difference in the height of the hammer before and after a fracture. The breaking energy (J) was recorded, calculated, and shown automatically on the digital display screen (see Figure 1). The energy absorption capacity (J/m^2^) was determined by dividing the breaking energy by the area of the failure surface. It is worth noting that, in this present work, the failure occurred at the notched sections. Therefore, the failure surface area was assumed as the area of the notched sections. Five samples of each P/C ratio were carried out for the Charpy test at 7, 14, and 28 days of curing.

In addition, the compressive strength test was conducted by means of a hydraulic universal testing machine with a capacity of 1000 kN, as presented in Figure 2. The test was performed in compliance with the ASTM C109/109M, and 3 specimens for each P/C ratio were examined at the curing age of 28 days. In summarization, 15 cube and 75 v-notched bar specimens were tested in this present study.

## 3. Results and Discussion

### 3.1. Compressive Strength

**Figure 3:**
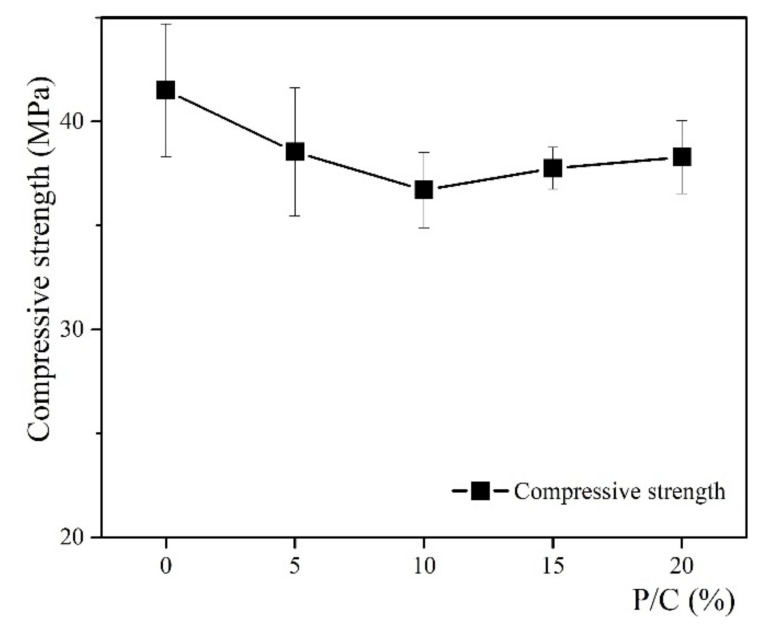
Compressive strength of the SBR latex-modified cement paste at 28 days.

**Figure 4:**
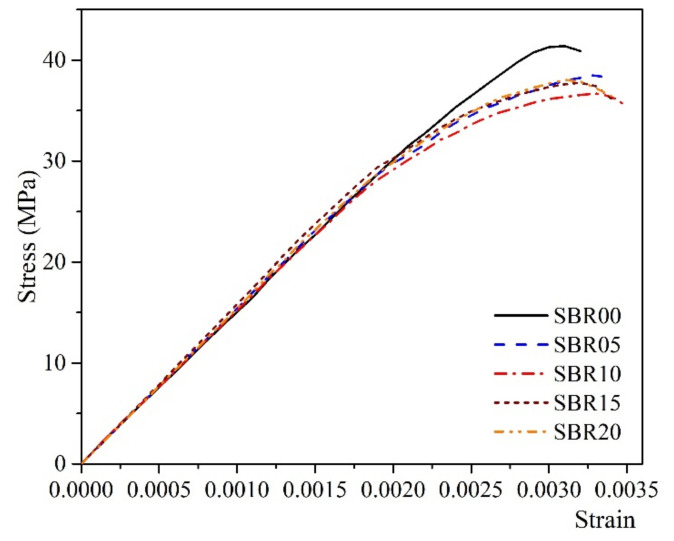
Constitutive curves of the SBR latex-modified cement paste at 28 days.

The compressive strength results of the 28-day OPC modified by SBR latex with different P/C ratios are given in Figure 3 and Figure 4, and Table 3.

As can be seen from Figure 3, with P/C ratios less than 10%, the compressive strength tended to decrease. In contrast, the compressive strength showed a slight increase with the P/C ratios higher than 10%. However, the compressive strength of the modified paste was lower than that of the control paste. Compared to the result of the control paste, there was a decrease of 12% in the compressive strength when modifying cement paste by the P/C of 10%. From Figure 4 and Table 3, the benefit of adding polymer into the cement paste is shown, as it increases the strain of the hardened cement paste and leads to an increase in the toughness of the cementitious materials. According to the theory of Timoshenko [35], toughness can be calculated by the area under the constitutive curve of the material. Following that approach, the results from Table 3 show that an increase in toughness corresponded with an increase in P/C ratios less than 10%, and with P/C ratios higher than 10%, the toughness slowly decreased. The observations from the compressive strength test at 28 days showed an agreement with the findings from the works of Wang et al. [31,32] on SBR latex-modified mortar. As demonstrated from their works, the mechanical properties of the polymer-modified cement-based materials are dependent on their phase states. The microstructure of the matrix changes when the polymer films are formed and when the hydration process is operated. In that case, the interpenetrating structure of the matrix is formed, which increases the matrix, restrains the tiny cracks, and leads to an improvement in the toughness of the matrix. However, when the interpenetrating structure is fully formed and the polymer film becomes thicker, the higher P/C ratios do not show their role in further improvement of the matrix properties. This can be pointed out with the P/C ratio of 10%. It is also worth mentioning that the SBR film has a very high toughness itself, thus the addition of SBR latex can contribute to improving the toughness of the matrix. As also observed from the works of Wang et al., SBR latex has lower strength compared to that of cement-based materials. In addition, adding SBR into cement-based materials increases the pore structure of the matrix and then reduces the bulk density. The increase in the ettringite content and decrease in calcium hydroxide content for the P/C ratios higher than 10% led to an increase in the strength of the paste [26]. However, the complexity of the changes in the microstructure of the matrix, such as an increase in the porosity or thickening of the polymer film, or even adding the polymer, also affects the cement hydration. Thus it might reduce the result of the matrix strength.

### 3.2. Impact Resistance

**Figure 5:**
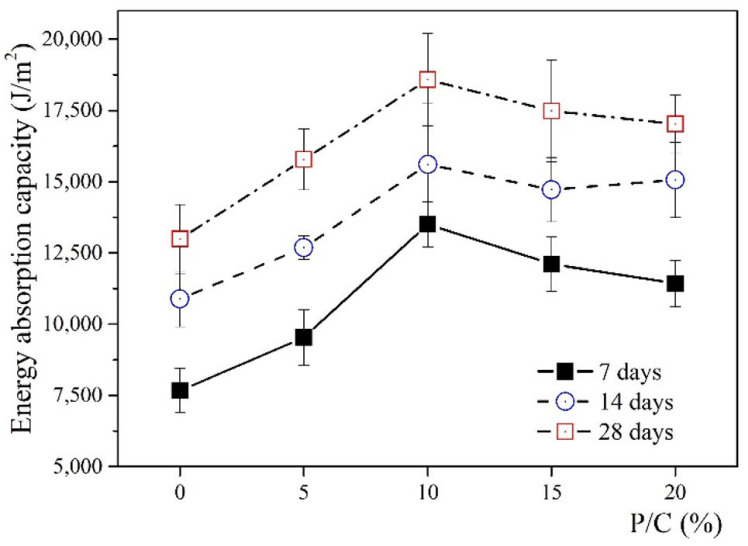
Influence of SBR latex on the energy absorption capacity of the cement paste at different ages of curing.

Due to the completely broken states of all specimens after testing, the observations from the Charpy test were acceptable. Figure 5 and Table 3 show the results of the energy absorption capacity (J/m^2^) obtained from the average value of five specimens for each P/C ratio. From an overall perspective, the energy absorption capacity of the SBR latex-modified cement paste increases with the increase in curing time. The time and the wet curing conditions might affect the creation of the polymer film as well as the hydration process of cement. A significant increase in energy absorption capacity of the SBR latex-modified cement paste was observed with the P/C ratio increase from 0 to 10% over the three curing ages of the study. The observations from 7 days showed a significant increase in the energy absorption capacity of the pastes containing the P/C ratios from 0 to 10%, then a decreasing trend was observed for that of the pastes containing the P/C ratios from 10 to 20%. For instance, the increases of 24, 76, 58, 49% were observed for the pastes containing the P/C ratios from 5 to 20%, respectively. At 14 days, the energy absorption capacity showed a similar trend with that at 7 days for the pastes containing the P/C ratios from 0 to 10%. In contrast, a slight decrease was observed when the P/C ratio was from 10 to 15%, then increased slightly from 15 to 20%. The increases of 17, 43, 35, 38% were reported for the pastes contain the P/C ratios from 5 to 20%, respectively. As can be observed from the result at 28 days, using the SBR latex in modifying cement paste showed its benefit by improving the energy absorption capacity of this material. There was a significant increase in the energy absorption capacity when the P/C ratios increased from 0 to 10%, and the observation showed an increase of 21 and 43% with the P/C ratio from 5 and 10%, respectively, compared to that of the control paste. Then the slight decreases of 43, 34, and 31% for the pastes containing the P/C ratios of 10, 15, and 20% were observed, respectively.

The observations from the Charpy test accord with the results of toughness as presented above. They increase when the P/C ratio increases from 0 to 10%, and with the other P/C ratios higher than 10%, there are no further improvements. The P/C ratio of 10% can be seen as an optimal ratio for observing the highest energy absorption capacity as well as toughness of SBR latex-modified cement paste.

**Figure 6:**
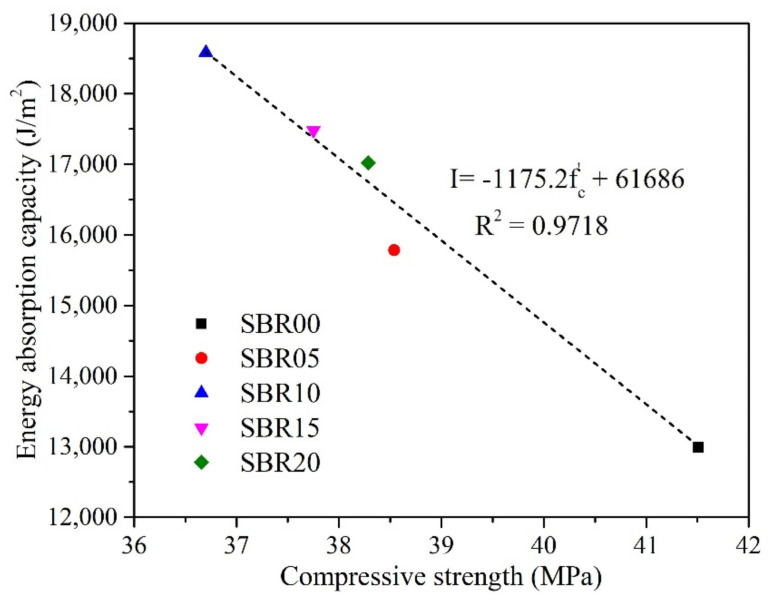
Relationship between compressive strength and energy absorption capacity of SBR latex-modified cement paste at 28 days.

As a comparison with the above observations at 28 days, with the increase in the P/C ratio from 0 to 10%, there is an increase in the energy absorption capacity and a decrease in the compressive strength. In contrast, with the other P/C ratios higher than 10%, an inversed tendency is observed. Therefore, it can be inferred that there is an inversed relationship between the energy absorption capacity and the compressive strength of the SBR latex-modified cement paste at the age of 28 days. The relationship between them is shown in Figure 6 with a coefficient of determination (R^2^) of 0.9718. Where I and f^’^_c_ are the energy absorption capacity (J/m^2^) and the compressive strength at 28 days (MPa) of the SBR latex-modified cement paste, respectively.

### 3.3. Weibull Distribution Analysis of the Charpy Impact Test

The literature review shows that the result from the impact test fit with the two-parameter Weibull distribution [36,37]. Following that approach, in this study, the two-parameter Weibull distribution was utilized to examine the reliability of the results from the Charpy impact test.

From [38], the probability distribution function *f*(*x*) and the cumulative density function *F_X_*(*x*) are denoted as below:

(1) $$f\left(x\right)=\frac{k}{u-\varepsilon }{\left(\frac{x-\varepsilon }{u-\varepsilon }\right)}^{k-1}e^{-{\left(\frac{x-\varepsilon }{u-\varepsilon }\right)}^{k}}$$

(2) $$F_{X}(x)=1-e^{-{\left(\frac{x-\varepsilon }{u-\varepsilon }\right)}^{k}}$$

where *x* is the specific value of random variables (in this present work it is the energy absorption capacity-I); *k* is the shape parameter or Weibull slope; *u* is the scale parameter; *ε* is the location parameter or minimum life. Assuming that *ε* = 0 for impact application [37], Equations (1) and (2) become:

(3) $$f\left(x\right)=\frac{k}{u}{\left(\frac{x}{u}\right)}^{k-1}e^{-{\left(\frac{x}{u}\right)}^{k}}$$

(4) $$F_{X}(x)=1-e^{-{\left(\frac{x}{u}\right)}^{k}}$$

The probability of survivorship is given by [38]:

(5) $$L_{X}\left(x\right)=1-F_{X}\left(x\right)=e^{-{\left(\frac{x}{u}\right)}^{k}}$$

Taking logarithms of both sides of Equation (5) gives.

(6) $$\ln\left[\ln\left(\frac{1}{L_{X}}\right)\right]=k\ln\left(x\right)-k\ln\left(u\right)$$

Therefore, Equation (6) can be used for verifying the statistical distribution of the energy absorption capacity (I) of three studied groups of specimens following the two-parameter Weibull distribution. The data of energy absorption capacity were sorted from smallest to largest and the empirical survivorship function can be obtained as:

(7) $$L_{X}=1-\frac{i}{N+1}$$

where *i* is the order number and *N* is the total number of specimens for each studied group.

Following the works [36,37], the linear relationship between ln[ln(1/*L_X_*)] and ln*x* showed the suitability of utilizing the two-parameter Weibull distribution to create statistical data of the energy absorption capacity. Then, the regression coefficients (*k*, *k*ln*u*) and the correlation coefficient R2 can be derived by linear analysis.

**Figure 7:**
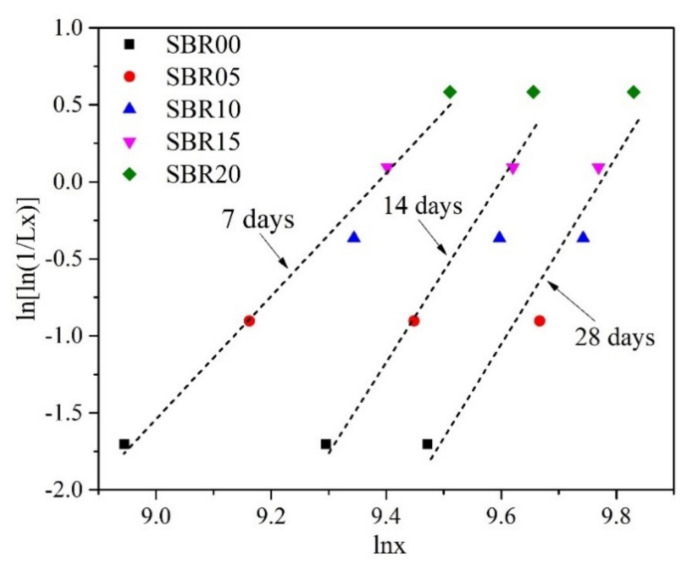
Weibull distribution of the energy absorption capacity (I).

Figure 7 and Table 4 show the variation in ln[ln(1/*L_X_*)] and lnx for three groups of cement paste specimens with different curing times. As in the above discussion, the linear relationship between ln[ln(1/*L_X_*)] and ln*x* is clarified. As a result, the two-parameter Weibull distribution is suitable for the description of the energy absorption capacity from the Charpy impact test.

## 4. Conclusions

The influence of SBR latex polymer with different P/C ratios on the energy absorption capacity of ordinary Portland cement was investigated in this study. The following conclusions may be drawn:

With P/C ratios less than 10%, increases in toughness and decreases in compressive strength are observed. In contrast, with P/C ratios over 10%, the observations show an inversed tendency.

The energy absorption capacity of the SBR latex-modified cement paste increases with the increase in the curing times from 7, 14, and, 28 days, as well as with the increase in the P/C ratios from 0, 5, 10, 15, and 20%. The energy absorption capacity of the SBR latex-modified cement paste increases significantly when the P/C ratio increases from 0 to 10%. With other P/C ratios higher than 10%, there are no further improvements. The P/C ratio of 10% is the optimal ratio for observing the highest energy absorption capacity of the SBR latex-modified cement paste, i.e., an increase of 43% compared to that of the control paste is observed.

An equation is found to describe the inversed relationship between the energy absorption capacity and the compressive strength of the SBR latex-modified cement paste at 28 days. As a result, the energy absorption capacity of the SBR latex-modified cement paste at 28 days can be analyzed or predicted by the compressive strength results, regardless of the P/C ratios.

The energy absorption capacity data observed from the Charpy impact test are fit with the two-parameter Weibull distribution.

The observations from this study can be a good reference for applying the SBR latex polymer-modified cement material for impact-resisting members, such as railway sleepers, blast-resisting members, cover members, and so on.

## Figures and Tables

**Table 1:** Physical properties of SBR latex.

TSC (%)	pH	Sp.gr (Kg/m^2^)	Viscosity (cps)	Average Particle Size (Å)	Tg (°C)	MFT (°C)
47.0	9–11	1090	<500	1500	−1	−1

TSC: total solid content; Sp.gr: specific gravity; T_g_: glass transition temperatures; MFT: minimum film-formation temperature.

**Table 2:** Mixture design by mass ratio.

Name	SBR00	SBR05	SBR10	SBR15	SBR20
Cement	1	1	1	1	1
SBR polymer (solid content)	0	0.05	0.1	0.15	0.2
Water	0.4	0.4	0.4	0.4	0.4

**Table 3:** Results from the compressive strength test and the Charpy impact test at 28 days.

Name	SBR00	SBR05	SBR10	SBR15	SBR20
Compressive strength (MPa)	41.51	38.54	36.7	37.75	38.29
	(3.191)	(3.077)	(1.801)	(1.005)	(1.743)
Toughness (J/m^3^)	74,725.0	79,970.2	81,586.6	80,797.6	80,138.7
Energy absorption capacity (J/m^2^)	12,991.5	14,848.1	18,582	17,483.1	17,021.5
	(778.1)	(982.1)	(792)	(956.7)	(816.3)

The values in parentheses are standard deviation.

**Table 4:** Linear regression coefficients of energy absorption capacity of the Weibull distribution.

Curing Time (days)	Regression Coefficient (*k*)	Regression Coefficient (*k*ln*u*)	Correlation Coefficient (R^2^)
7	3.951	37.092	0.984
14	5.724	54.971	0.938
28	6.224	60.805	0.942

## Data Availability

The data presented in this study are available on request from the corresponding author.
